# The MICOS Complex Subunit Mic60 is Hijacked by Intracellular Bacteria to Manipulate Mitochondrial Dynamics and Promote Bacterial Pathogenicity

**DOI:** 10.1002/advs.202406760

**Published:** 2024-10-21

**Authors:** Changyong Cheng, Mianmian Chen, Jing Sun, Jiali Xu, Simin Deng, Jing Xia, Yue Han, Xian Zhang, Jing Wang, Lei Lei, Ruidong Zhai, Qin Wu, Weihuan Fang, Houhui Song

**Affiliations:** ^1^ Key Laboratory of Applied Technology on Green‐Eco‐Healthy Animal Husbandry of Zhejiang Province Zhejiang Provincial Engineering Research Center for Animal Health Diagnostics & Advanced Technology Zhejiang International Science and Technology Cooperation Base for Veterinary Medicine and Health Management China‐Australia Joint Laboratory for Animal Health Big Data Analytics College of Veterinary Medicine of Zhejiang A&F University 666 Wusu Street, Lin'an District Hangzhou Zhejiang Province 311300 China

**Keywords:** host‐microbe interplay, listeriolysin O (LLO), Mic60, MICOS, mitochondrial fragmentation

## Abstract

Host mitochondria undergo fission and fusion, which bacteria often exploit for their infections. In this study, the underlying molecular mechanisms are aimed to clarify through which *Listeria monocytogenes* (*L. monocytogenes*), a human bacterial pathogen, manipulates mitochondrial dynamics to enhance its pathogenicity. It is demonstrated that *L. monocytogenes* triggers transient mitochondrial fission through its virulence factor listeriolysin O (LLO), driven by LLO's interaction with Mic60, a core component of the mitochondrial contact site and the cristae organizing system (MICOS). Specifically, Phe251 within LLO is identify as a crucial residue for binding to Mic60, crucial for LLO‐induced mitochondrial fragmentation and bacterial pathogenicity. Importantly, it is that Mic60 affect the formation of F‐actin tails recruited by *L. monocytogenes*, thereby contributing to intracellular bacterial infection. Mic60 plays a critical role in mediating changes in mitochondrial morphology, membrane potential, and reactive oxidative species (ROS) production, and *L. monocytogenes* infection exacerbates these changes by affecting Mic60 expression. These findings unveil a novel mechanism through which intracellular bacteria exploit host mitochondria, shedding light on the complex interplay between hosts and microbes during infections. This knowledge holds promise for developing innovative strategies to combat bacterial infections.

## Graphical abstract

Upon the invasion of Listeria monocytogenes into a host cell, the primary virulence factor, listeriolysin O (LLO), secreted by the bacterium, has the capacity to breach the mitochondrial membrane, thereby gaining access to the inner mitochondrial membrane (IMM). The interaction between LLO and Mic60 can induce significant changes in the architecture of the inner mitochondrial membrane, leading to alterations in mitochondrial dynamics and function, which in turn, can favor the bacterial infection process.

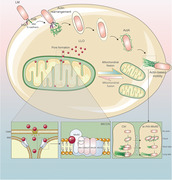

## 1 Introduction

Mitochondria, the powerhouses of the cells, are highly dynamic organelles that are essential for multiple metabolic processes, including the production of energy, oxygen sensing, and induction of innate immune responses.^[^ ^1^, ^2^ ^]^ Mitochondrial dynamics refers mostly to mitochondrial morphological alteration, mainly comprising of fusion and fission.^[^ ^3^ ^]^ Mitochondrial fission is characterized by the division of one mitochondrion into two daughter mitochondria, whereas mitochondrial fusion is the union of two mitochondria resulting in one mitochondrion.^[^ ^4^ ^]^ The steady‐state balance between fusion and fission is crucial for maintaining a healthy and functional mitochondrial network.^[^ ^5^ ^]^ Mitochondria undergo dynamics through the action of GTPases that belong to the dynamin family to ensure mitochondrial membrane remodeling, including mitofusins 1 and 2 (Mfn1 and Mfn2) and optic atrophy 1 (Opa1), which mediate outer mitochondrial membrane (OMM) and inner mitochondrial membrane (IMM) fusion,^[^ ^6^ ^]^ respectively. Moreover, mitochondrial fission is mediated mainly by dynamin‐related protein 1 (Drp1) and dynamin 2 (Dnm2), forming ring‐like structures to constrict mitochondria.^[^ ^7^ ^]^

Considering the critical functions of mitochondria, many intracellular bacteria have evolved mechanisms to disturb mitochondrial homeostasis by manipulating the mitochondrial dynamics to favor their benefits during infection.^[^ ^8^, ^9^, ^10^, ^11^ ^]^ Indeed, *Listeria monocytogenes* (*L. monocytogenes*), the causative bacterial pathogen of human listeriosis,^[^ ^12^ ^]^ can interfere with mitochondrial dynamics and induce a strong and rapid but transient fragmentation of the mitochondrial network at early infection (within one hour) in epithelial cells. This event requires the bacterial pore‐forming toxin listeriolysin O (LLO) that belongs to the family of cholesterol‐dependent cytolysins (CDCs).^[^ ^13^, ^14^ ^]^ Besides, a very recent study showed that *L. monocytogenes* infection significantly upregulates the mitochondrial levels of Mic10,^[^ ^15^ ^]^ a core subunit of the mitochondrial contact site and cristae organization system (MICOS) that is essential for the formation and maintenance of mitochondrial cristae structure.^[^ ^16^, ^17^ ^]^ Moreover, Mic10 was found necessary for *L. monocytogenes* infection‐induced mitochondrial fragmentation.^[^ ^15^ ^]^ However, the mechanistic correlation of LLO with MICOS, as well as the underlying basis in *L. monocytogenes* infection‐induced mitochondrial dynamics, needs to be elucidated, and it remains unclear if there are any other virulence factors involved in this process.

In this study, we identified the MICOS core component, Mic60, as the host factor that can be targeted by LLO through protein‐to‐protein interactions using the co‐immunoprecipitation, and the residue Phe251 is critical forbinding of LLO to Mic60 and required for LLO‐triggered mitochondrial fragmentation. Interestingly, we further discovered that the endogenous Mic60 is required for bacterial infection‐triggered mitochondrial network fragmentation and contributes to actin‐based intracellular bacterial infection of host cells since knockdown or overexpression of Mic60 significantly decreased the ability of *L. monocytogenes* to grow intracellularly. Our findings collectively provided a novel insight into the molecular machinery exploited by intracellular bacteria to manipulate host mitochondrial dynamics and facilitate their infections.

## 2 Results

### 2.1 Mitochondrial Fragmentation is Triggered by LL0

**Figure 1:**
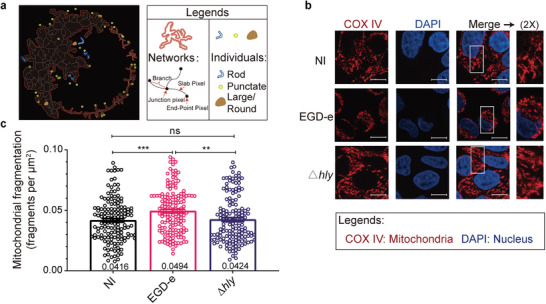
Mitochondrial fragmentation is triggered by LLO. a) Diagram of quantification for mitochondrial network morphology using ImageJ plugin tool MiNA on mitochondrion labeled images. The mitochondrial fragmentation degree was calculated by using the values listed under “Individuals” normalized to “Mitochondrial footprint” (mitochondrial area). The left panel is the model of mitochondria structure. The right panel is the legend of the left panel. The normal structure of mitochondria contains networks and individuals. The mitochondrial network has many junctions and branches. Individuals include punctate, rod, large/round. Punctate is a single point, rod is a branchless structure and large/round is a large or circular structure. The mitochondrial footprint (mitochondrial area) is the total area of networks and individuals. b) Immunofluorescence analysis of HeLa cells infected for 2 h with wild‐type, and △*hly L. monocytogenes* at an MOI of 50. Mitochondria are shown in red (COX IV) and nuclei in blue (DAPI). The white box indicates a region of the mitochondrial network magnified (2x) in the inset shown at the right. NI, no infection; Scale bars, 10 µm. c) Quantification for the degree of mitochondrial fragmentation in HeLa cells infected with *L. monocytogenes*. Scatter plots show fragmentation degree values for each cell (at least 50 dots), and data are expressed as means ± SE of three independent experiments. NI, no infection; **, *p* < 0.01; ***, *p* < 0.001.

It has been reported that *L. monocytogenes* infection‐induced mitochondrial fragmentation depends on the pore‐forming toxin LLO.^[^ ^13^, ^14^ ^]^ We verified whether LLO could manipulate mitochondrial dynamics during infection. We used a semiautomated morphometric tool (Image J) to quantify the mitochondrial networks or individuals of a large number of cells,^[^ ^15^ ^]^ which allowed us to determine the degree of mitochondrial fragmentation (**Figure** **1**a). We used wild‐type *L. monocytogenes* and △*hly* to infect HeLa cells for 2 hours and then analyzed how the mitochondrial network morphology was affected. In agreement with previous results, infected cells showed a typical mitochondrial network that fragmented upon infection with wild‐type EGD‐e but not *hly*‐deficient (△*hly*) bacteria (Figure 1b). The data confirmed the increased mitochondrial fragmentation in cells infected with wild‐type *L. monocytogenes* compared to cells infected with △*hly* (Figure 1c), revealing that LLO, is required for fragmentation of host‐cell mitochondria during bacterial infection.

### 2.2 LLO Interacts with Host MICOS Core Subunit, Mic60, at the Residue Phe251 that is Required for LLO‐Induced Mitochondrial Fragmentation

**Figure 2:**
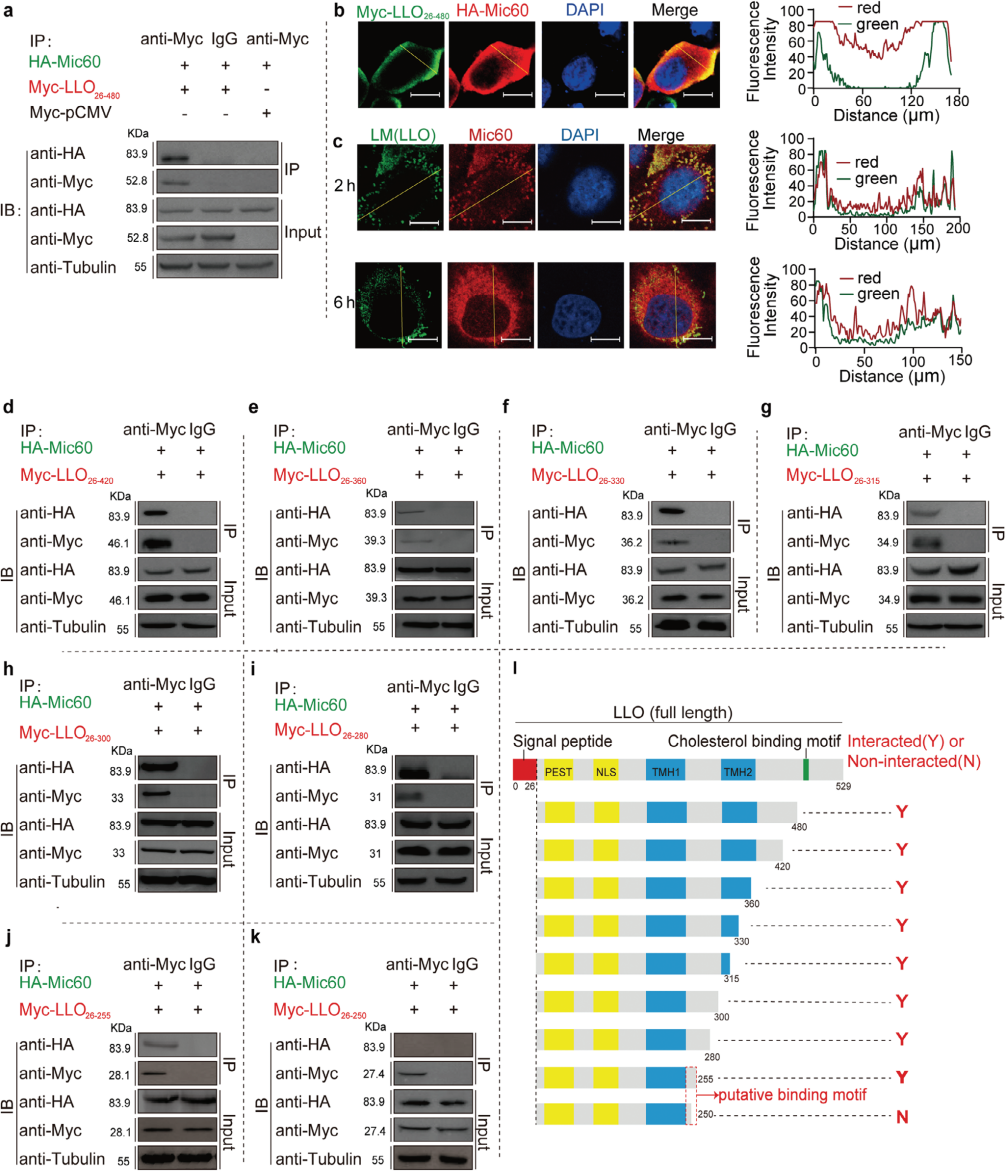
The MICOS core subunit Mic60 is the LLO‐interacting target. a) Co‐immunoprecipitation of HA‐Mic60 with Myc‐LLO from co‐transfected HEK293T cells using an anti‐Myc antibody. The anti‐IgG was used as a negative control for immunoprecipitation. Subsequent immunoblotting was performed using antibodies against HA and Myc tags. Tubulin protein was used as the internal control. b) Fluorescence colocalization analysis of HA‐Mic60 with Myc‐LLO from co‐transfected HEK293T cells. At 24 hours post‐transfection, cells were permeabilized and stained with anti‐Myc (red), anti‐HA antibodies (green), and nuclear dye DAPI (blue). Scale bars, 10 µm. Quantitative analyses of colocalizations were performed with ImageJ software. c) Fluorescence colocalization analysis of endogenous Mic60 with LLO produced from *L. monocytogenes* infection in HeLa cells. At 2 or 6 hours post bacterial infection, cells were washed, permeabilized, and stained with anti‐Mic60 (red), anti‐LLO antibodies (green), and nuclear dye DAPI (blue). Scale bars, 10 µm. d–k) Co‐immunoprecipitation of HA‐Mic60 with Myc tagged LLO truncation derivatives from co‐transfected HEK293T cells using an anti‐Myc antibody. The anti‐IgG was used as a negative control for immunoprecipitation. Subsequent immunoblotting was performed using antibodies against HA and Myc tags. Tubulin protein was used as the internal control. l Schematic diagrams of different LLO truncations constructs that were used being tested in the co‐IP assay. NLS is a putative nuclear localization sequence, PEST is involved in bacterial phagosome escape in infected cells and is critical for bacterial virulence, and TMH1 and TMH2 are transmembrane helixes.

**Figure 3:**
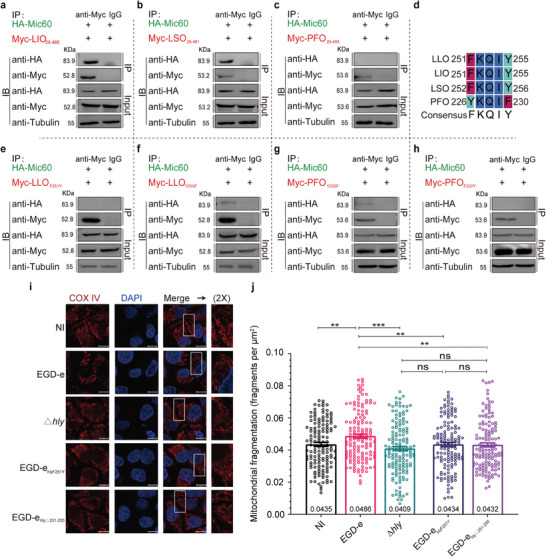
The residue Phe251 is required for the interaction of LLO with Mic60 and LLO‐dependent mitochondrial fragmentation. a‐c Co‐immunoprecipitation of Mic60 with *L. innocua* LIO a) *L. seeligeri* LSO b), or C. *perfringens* PFO c) from co‐transfected HEK293T cells using an anti‐Myc antibody. The anti‐IgG was used as a negative control for immunoprecipitation. Subsequent immunoblotting was performed using antibodies against HA and Myc tags. Tubulin protein was used as the internal control. d) Alignment of the identified binding pentapeptide (F_251_KQIY_255_) of LLO to those homologs from LIO, LSO, and PFO. e‐h Co‐immunoprecipitation of Mic60 with LLO_F251Y_ e), LLO_Y255F_ f), PFO_Y226F_ g), or PFO_F230Y_ h) from co‐transfected HEK293T cells using an anti‐Myc antibody. The anti‐IgG was used as a negative control for immunoprecipitation. Subsequent immunoblotting was performed using antibodies against HA and Myc tags. Tubulin protein was used as the internal control. i) Immunofluorescence analysis of HeLa cells infected for 2 h with wild‐type, △*hly*, *hly*_F251Y_ or, *hly*△_251‐255_ *L. monocytogenes* at an MOI of 50. Mitochondria are shown in red (COX IV) and nuclei in blue (DAPI). The white box indicates a region of the mitochondrial network magnified (2x) in the inset shown at the right. NI, no infection; Scale bars, 10 µm. j) Quantification for the degree of mitochondrial fragmentation in HeLa cells infected with wild‐type, △*hly*, *hly*_F251Y_ or, *hly*△_251‐255_ *L. monocytogenes*. Scatter plots show fragmentation degree values for each cell (at least 50 dots), and data are expressed as means ± SE of three independent experiments. NI, no infection; ns, not significant; **, *p* < 0.01; ***, *p* < 0.001.

In our preliminary research, we discovered that phospholipase C (PlcB), a virulence factor secreted alongside LLO by *L. monocytogenes*, also influences mitochondrial dynamics during infection. To examine possible interactions between this bacterial protein and mitochondria, we employed a version of PlcB lacking its signal peptide to probe a normalized yeast two‐hybrid (Y2H) library containing fragmented human cDNAs (Figure S1a, Supporting Information). This led to the identification of the mitochondrial inner membrane protein Mic60 (also known as IMMT or Mitofilin) as a direct interaction partner of PlcB (Figure S1b, Supporting Information). Considering LLO's significant role in mitochondrial fragmentation, we further investigated its relationship with Mic60 using co‐immunoprecipitation without LLO's signal peptide. Our findings confirmed Mic60 interacts with LLO (**Figure** **2**a). Subsequent immunofluorescence assays in transfected HEK293T cells revealed co‐localization of Myc‐tagged LLO with HA‐tagged Mic60 (Figure 2b), and during *L. monocytogenes* infection within HeLa cells, intracellular LLO was found to colocalize significantly with endogenous Mic60, forming punctate structures around the nucleus (Figure 2c). Furthermore, LLO was also found to interact with the endogenous Mic60 in mitochondria following bacterial infection (Figure S2, Supporting Information). Moreover, co‐immunoprecipitation of Mic60 with LLO truncation derivatives revealed that the pentapeptide (251‐255, FKQIY) in LLO was the region required for binding with Mic60 (Figure 2d–l). To test whether this interaction is the unique feature of LLO amongst CDCs, two other *Listeria* species LLO homologs (LIO and LSO from *L. innocua* and *L. seeligeri*, respectively) as well as another CDC toxin, perfringolysin O (PFO) from *Clostridium perfringens*, were selected for studying the interaction with Mic60. As shown by the immunoprecipitation assays, Mic60 was able to interact with LIO or LSO in transfected HEK293T cells but unable to interact with PFO (**Figure** **3**a–c). The identified binding pentapeptide FKQIY of LLO was then aligned from its homologs, showing that this region was utterly conserved in LIO and LSO. At the same time, there exist two substitutions in the corresponding region of PFO (Y226 and F230) compared with those of LLO (F251 and Y255) (Figure 3d). To examine whether these two substitutions could determine the ability of LLO or the inability of PFO to interact with Mic60, four Myc‐tagged LLO mutants (LLO_F251Y_ and LLO_Y255F_) and PFO mutants (PFO_Y226F_ and PFO_F230Y_) were constructed and then subjected to co‐immunoprecipitation with Mic60 in transfected HEK293T cells. Strikingly, we showed that the substitution F251Y rendered LLO no longer capable of interacting with Mic60; however, the Y255F mutation in LLO retains its ability to interact with Mic60 (Figure 3e,f), revealing that F251 is necessarily required for binding of LLO to Mic60. Moreover, it is the substitution Y226F, but not F230Y, that makes PFO able to interact with Mic60 (Figure 3g,h), further confirming the essential role for the residue F251 in the binding of LLO to Mic60. Then, the substitution F251Y was introduced into the wild‐type chromosome to explore the roles of F251 in LLO‐induced mitochondrial fragmentation. As expected, the cells infected with the *hly*_F251Y_ or *hly*_△251‐255_ mutant exhibited significantly decreased mitochondrial fragmentation compared with cells infected with the wild‐type (Figure 3i,j). These comprehensive findings illustrate a complex interaction landscape where LLO targets host mitochondria through Mic60, influencing mitochondrial dynamics.

### 2.3 Phe251 of LLO is Critical to Listeria Pathogenicity

**Figure 4:**
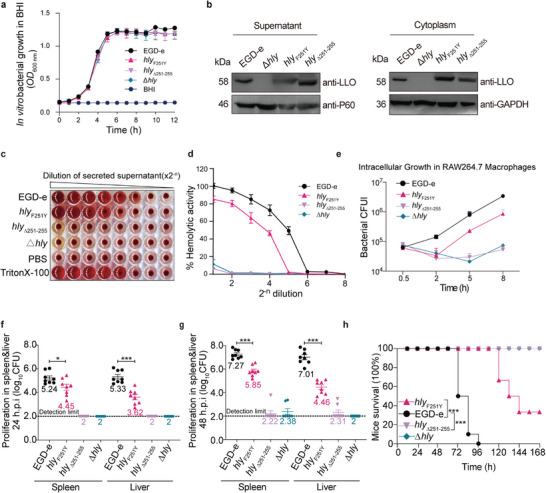
Phe251 of LLO is critical to *Listeria* pathogenicity. a) In vitro growth of wild‐type *L. monocytogenes*, △*hly*, *hly*_F251Y_, or *hly*_△251‐255_ in BHI broth. b) Secreted and cellular LLO was detected by Western blotting of *L. monocytogenes* wild‐type, △*hly*, *hly*_F251Y_ or, *hly*_△251‐255_. Immunoblotting was performed with antibodies against LLO. GAPDH and p60 were used as internal controls for the cellular and secreted protein samples, respectively. c,d) Hemolytic activity of secreted LLO from the culture supernatants of the wild‐type *L. monocytogenes*, △*hly*, *hly*_F251Y_, or *hly*_△251‐255_. Erythrocytes incubated with 1% Triton X‐100 or phosphate‐buffered saline (PBS) were used to determine the maximum (100%) and minimum (0%) hemolytic activity, respectively. e Intracellular growth of *L. monocytogenes* LLO mutants in RAW264.7 macrophages. Macrophages infected with the indicated strains were lysed at the indicated time points (0.5, 2, 5, and 8 h), and viable bacteria were serially diluted and plated on BHI agar. f‐g Proliferation of *L. monocytogenes* LLO mutants in mice livers and spleens. The wild‐type and mutant strains were inoculated intraperitoneally into ICR mice at ≈1 × 10^6^ CFU. Animals were euthanized at 24 f) or 48 h g) post infection, and organs were harvested and homogenized. Homogenates were serially diluted and plated on BHI agar. The numbers of bacteria colonizing the organs are expressed as means ± SDs of the log_10_ CFU per organ for each group. h Kaplan–Meier curve showing the survival of the ICR mice over time. Ten mice in each experimental group were infected intraperitoneally with 1 × 10^6^ CFU of *Listeria* and monitored for up to 7 days post‐infection. Data are presented as the percentage survival over time, and significance was determined by the log‐rank test. *, *P*<0.05; ***, *P*<0.001.

To further explore the potential roles of Phe251 in LLO‐mediated bacterial pathogenicity, we compared the abilities of the mutants *hly*_△251‐255_ and *hly*_F251Y_ in LLO production and bacterial infection. First, the *hly*_△251‐255_ and *hly*_F251Y_ mutants showed normal growth in vitro as the wild‐type (**Figure** **4**a). Also, as shown by western blotting, these two mutants were capable of expressing and secreting LLO of comparable amounts to the wild‐type LLO, suggesting these mutations did not affect LLO production (Figure 4b). Also, we discovered that the *hly*_F251Y_ exhibited slightly decreased hemolytic activity compared to those of the wild‐type strain (Figure 4c,d). However, similar to the △*hly* mutant, the *hly*_△251‐255_ did not exhibit any detectable hemolytic activity (Figure 4c,d). We then investigated these mutants' ability to grow intracellularly in macrophages and proliferate in mouse organs. As indicated in Figure 4e, the intracellular growth was significantly impaired for the *hly*_F251Y_ and completely lost for the *hly*_△251‐255_. Moreover, the number of bacteria recovered from the spleens and livers of the infected mice after 24 and 48 h of infection was significantly lower (≈2.6‐log decrease in bacterial burden for the liver at 48 hours) for the *hly*_F251Y_ mutant compared with that for the wild‐type (Figure 4f,g). For the *hly*_△251‐255_ mutant, almost no detectable bacteria were recovered from the mice organs 24 and 48 hpi, which is similar to the circumstance in the avirulent △*hly* mutant (Figure 4f,g). Additionally, infection of *hly*_F251Y_ or *hly*_△251‐255_ resulted in 30% or 100% survival of the mice, respectively, at 7 days post infection, comparing with the wild‐type leading to 100% mortality at ≤4 days post infection (Figure 4h), indicating these two mutants were severely attenuated for virulence. Taken together, these results demonstrate an essential role for the residue Phe251 of LLO in the pathogenicity of *Listeria*.

### 2.4 Mic60 Contributes to *L. Monocytogenes*‐Mediated Mitochondrial Fragmentation

**Figure 5:**
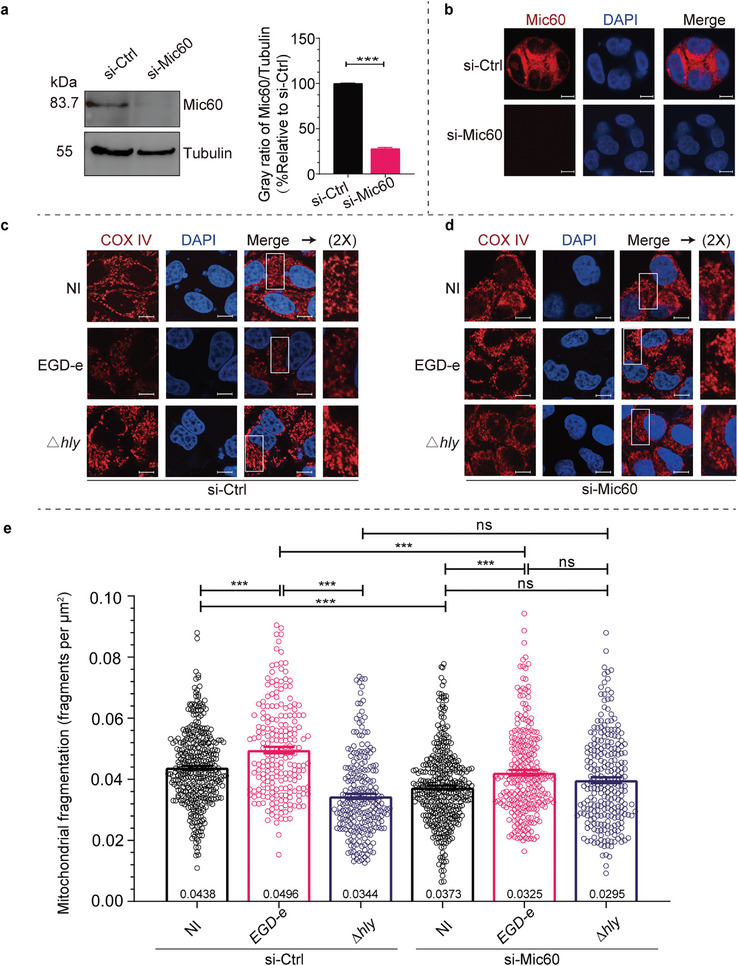
Mic60 knockdown decreases *L. monocytogenes* infection‐induced mitochondrial fragmentation. a) Immunoblotting analysis of endogenous Mic60 levels in HeLa cells transfected with nontargeting (si‐Ctrl) or Mic60‐targeting (si‐Mic60) siRNAs. Tubulin protein was used as the internal control. The histogram on the right indicates the grayscale ratio of Mic60 to tubulin, with the control group set to 100%. Data are expressed as means ± SE of three independent experiments. **, *p* < 0.01. b) Immunofluorescence analysis of HeLa cells transfected with si‐Ctrl or si‐Mic60 siRNAs. Mic60 proteins are shown in red (anti‐Mic60) and nuclei in blue (DAPI). Scale bars, 10 µm. c,d) Immunofluorescence analysis of HeLa cells transfected with si‐Ctrl (c) or si‐Mic60 (d) siRNAs, which were infected for 2 h with wild‐type, △*hly L. monocytogenes* at an MOI of 50. Mitochondria are shown in red (COX IV) and nuclei in blue (DAPI). The white box indicates a region of the mitochondrial network magnified (2x) in the inset shown at the right. NI, no infection; Scale bars, 10 µm. e) Quantification for the degree of mitochondrial fragmentation in HeLa cells infected with *L. monocytogenes*. Scatter plots show fragmentation degree values for each cell (at least 50 dots), and data are expressed as means ± SE of three independent experiments. NI, no infection; ns, not significant; ***, *p* < 0.001.

As the fact is that Mic60 is essential for the integrity of MICOS complex and functions as a mediator linking mitochondrial outer membrane and inner membrane fusion or fission,^[^ ^17^, ^18^ ^]^ therefore we sought to explore the potential roles of Mic60 in *L. monocytogenes* infection‐induced mitochondrial fission. We firstly transfected Mic60‐targeting small interfering RNAs (si‐Mic60) to knockdown the endogenous Mic60 levels in HeLa cells prior to bacterial infection, before which the interfering effect was confirmed by western blot and immunofluorescence experiments using non‐targeting siRNAs as the control (si‐Ctrl) (**Figure** **5**a,b). Consistent with the previous report, ^(^ ^17^ ^)^ the Mic60 knockdown caused significantly decreased mitochondrial fragmentation in uninfected HeLa cells (Figure 5c–e). In agreement with our results mentioned above, cells treated with si‐Ctrl exhibited a typically mitochondrial network that fragmented much upon infection with wild‐type bacteria compared to uninfected si‐Ctrl cells or cells infected with △*hly* bacteria (Figure 5c). Similarly, in si‐Mic60 transfected cells, mitochondrial fragmentation was found to increase in cells infected with bacteria in comparison to uninfected si‐Mic60 cells. However, unlike the findings observed in si‐Ctrl cells, no significant changes in mitochondrial fragmentation were detected in si‐Mic60 cells infected with wild‐type *L. monocytogenes* compared to cells infected with △*hly* bacteria (Figure 5d). The findings were confirmed by an unbiased, quantitative analysis where the degree of mitochondrial fragmentation per cell is determined (Figure 5e). Next, we evaluated whether increasing host Mic60 expression would have potential effects on LLO‐mediated mitochondrial fragmentation during *L. monocytogenes* infection. Cells were transiently transfected with either a vector constitutively expressing an HA‐tagged human Mic60 protein (HA‐Mic60) or with the empty vector (pCMV) as a negative control. By using western blot and immunofluorescence analysis, we confirmed the presence of the HA‐tag associated with the exogenously expressed Mic60 from cells collected 24 hours post‐transfection. The analysis also confirmed the overexpression of total Mic60 in Mic60‐transfected cells and showed decreased endogenous Mic60 levels in Mic60‐transfected cells compared to control cells (Figure S3a,b, Supporting Information). Similar to the circumstance in Mic60‐depletion cells, we found that overexpression of Mic60 also resulted in a significant defect in mitochondrial fragmentation in uninfected cells (Figure S3c–e, Supporting Information), which was consistent with the previous findings in neuronal cells where overexpressing Mic60 displayed suppressed mitochondrial fission and increased mitochondrial length.^[^ ^18^ ^]^ Moreover, cells transfected with the empty vector exhibited significantly increased mitochondrial fission upon infection with wild‐type *L. monocytogenes* in comparison with uninfected cells or cells infected with *hly*‐deficient bacteria (Figure S3c,e, Supporting Information). However, cells overexpressing Mic60 showed a minor increased mitochondrial fragmentation upon infection with wild‐type bacteria compared to uninfected cells, meanwhile, there were apparent differences displayed among wild‐type *L. monocytogenes* and △*hly* (Figure S3d,e, Supporting Information). Collectively, these data revealed that the endogenous Mic60 appears necessary and sufficient for *L. monocytogenes* infection‐induced mitochondrial network fragmentation in LLO‐dependent manners.

### 2.5 Mic60 Contributes to Actin‐Based *L. Monocytogenes* Intracellular Infection

**Figure 6:**
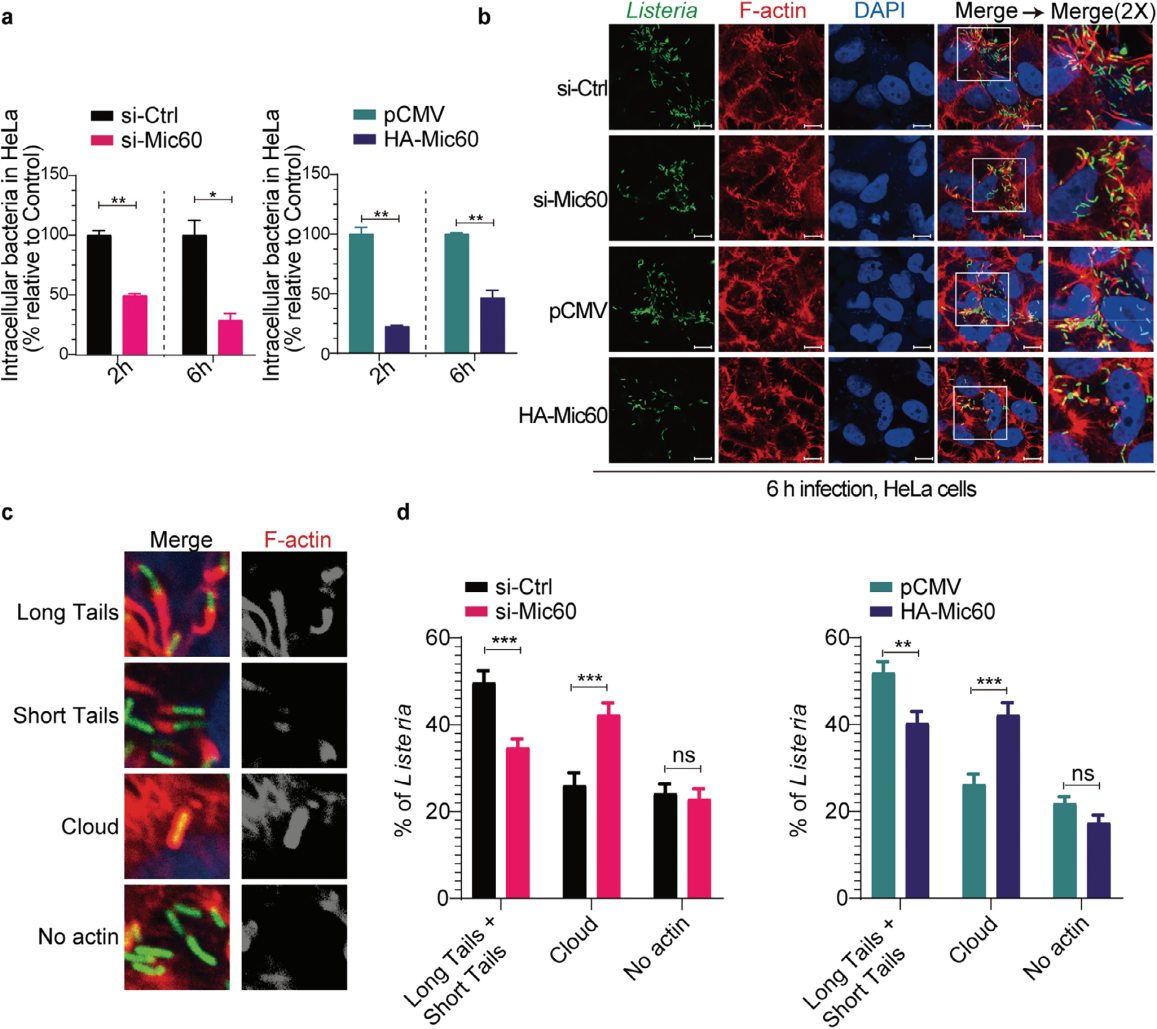
Mic60 correlates positively with *L. monocytogenes* intracellular infection. a) Intracellular bacteria in HeLa cells transfected with nontargeting control (si‐Ctrl) or Mic60‐targeting (si‐Mic60) siRNAs, or transiently transfected with the control plasmid (pCMV) or a plasmid constitutively expressing N‐terminal HA‐tagged Mic60 (HA‐Mic60), following infection with wild‐type *L. monocytogenes* for 2 or 6 hours. Data represent means ± SE from three independent experiments and are expressed as percentages of intracellular bacteria relative to those quantified in control cells. b) Immunofluorescence analysis of HeLa cells transfected with si‐Ctrl or si‐Mic60 siRNAs, or transiently transfected with pCMV or HA‐Mic60 plasmids, following infection with GFP‐expressing *L. monocytogenes* for 6 hours. F‐actin was shown in red (phalloidin‐Alexa Fluor 568), nuclei in blue (DAPI), and bacteria in green. Scale bars, 10 µm. c) Representative images of the actin tail pattern. Left panel: Merged image: green, Bacteria; red, F‐actin. Right panel: F‐actin only. d) Quantification of actin tail patterns as shown in (c) from HeLa cells transfected with si‐Ctrl or si‐Mic60 siRNAs, or transiently transfected with pCMV or HA‐Mic60 plasmids, following infection with GFP‐expressing *L. monocytogenes* for 6 hours. Data are expressed as means ± SE of three independent experiments. ns, not significant; *, *p* < 0.05; **, *p* < 0.01; ***, *p* < 0.001.

Having established that Mic60 is required for *L. monocytogenes*‐mediated mitochondrial fragmentation and combining a recent finding that another MICOS subunit Mic10 contributes to *L. monocytogenes* cellular infection,^[^ ^15^ ^]^ we thus tried to explore any potential roles that Mic60 would play in the process of intracellular *L. monocytogenes* infection. We compared the ability of wild‐type bacteria to grow intracellularly in HeLa cells depleted of Mic60 or overexpressing Mic60. As indicated, the Mic60‐knockdown cells showed about 50% less load of intracellular bacteria than si‐Ctrl cells after 2 or 6 hours infection, and more interestingly, cells overexpressing Mic60 appeared to have a more significant decrease (about 50%) in bacterial load compared to those cells transfected with the empty vector (**Figure** **6**a). To further investigate the possible roles of Mic60 in actin‐based *L. monocytogenes* spread, we analyzed actin tail pattern formation in Mic60‐depleted or Mic60‐overexpression HeLa cells during *L. monocytogenes* infection (Figure 6b). Actin tail pattern formation was evaluated by quantifying the percentage of bacteria that displayed long or short actin tails, or were surrounded by F‐actin (clouds), or were not associated with F‐actin (Figure 6c). Strikingly, we discovered that knockdown or overexpression of Mic60 resulted in a significant decrease in bacteria with long/short actin tails and an increase in bacteria surrounded by actin clouds, while not affecting the bacteria that displayed no tails (Figure 6d). These findings suggested that endogenous Mic60 strongly correlates with the recruitment of F‐actin by *L. monocytogenes* and therefore contributes to *L. monocytogenes* actin‐based spread during intracellular infection.

Generally, deletions of the core subunits of the MICOS complex have drastic effects on the architecture of the mitochondrial inner membrane, as well as on the overall mitochondrial morphology.^[^ ^19^, ^20^ ^]^ Specifically, Mic60 is critical for maintaining mitochondrial membrane structure and function, and knockdown of Mic60 causes the formation of “giant mitochondria” and is associated with an impaired mitochondrial respiration capacity.^[^ ^17^, ^18^ ^]^ Mic60 overexpression also suppresses mitochondrial fission and increases mitochondrial interconnectivity.^[^ ^18^ ^]^ Thus, we believe that Mic60‐deficient/‐overexpression cells fail to provide the replication niche for *L. monocytogenes* to establish its infection, which could account for the compromised ability of *L. monocytogenes* to grow intracellular in Mic60‐knockdown or Mic60‐overexpression cells. Taken together, we concluded that the rigorously controlled Mic60 as a crucial component of the MICOS complex contributes to the actin‐based intracellular infection of *Listeria*.

### 2.6 *L. Monocytogenes* Infection Exacerbates Mitochondrial Dysfunction

We further investigated the potential role of Mic60 in *L. monocytogenes* infection‐mediated changes in mitochondrial morphology, mitochondrial membrane potential (ΔΨ), and mitochondrial reactive oxidative species (ROS) production. Our findings indicated that wild‐type *L. monocytogenes* treatment caused a significant disruption of mitochondria ultrastructure compared to untreated cells or △*hly L. monocytogenes* treated cells (Figure S4a,d,e, Supporting Information). In addition, Mic60 knockdown cells exhibited severe disruption of mitochondrial ultrastructure, whereas Mic60 overexpression did not (Figure S4b,c, Supporting Information). The assessment of mitochondrial ROS production showed that Mic60 knockdown or Mic60 overexpression led to a significant increase in mitochondrial ROS production compared to control cells (Figure S4f, Supporting Information). Furthermore, wild‐type *L. monocytogenes* treatment further increased mitochondrial ROS production both in Mic60 knockdown and Mic60 overexpression cells (Figure S4f, Supporting Information). In the evaluation of mitochondrial membrane potential loss, Mic60 knockdown or Mic60 overexpression resulted in a significant decrease in ΔΨ compared to control cells. Wild‐type *L. monocytogenes* treatment further reduced ΔΨ in Mic60 knockdown cells (Figure S4g, Supporting Information). Mitochondrial ROS production and mitochondrial membrane potential showed similar levels in △*hly* and *hly*_F251Y_ in Mic60 knockdown cells, but not in Mic60 overexpression cells. In summary, our results indicate that Mic60 plays a critical role in mediating changes in mitochondrial morphology, mitochondrial membrane potential, and mitochondrial ROS production, and that *L. monocytogenes* infection exacerbates these changes, suggesting that *L. monocytogenes* causes structural and functional damage to mitochondria.

## 3 Discussion

Mitochondria are dynamic organelles whose morphology is determined by continuous fusion and fission events.^[^ ^21^ ^]^ Moreover, increasingly emerging research has substantiated that mitochondria serve as hubs for innate immune signaling against bacteria,^[^ ^22^ ^]^ making mitochondria very attractive structures to be targeted and modulated by bacterial pathogens to favor their infection^[^ ^8^, ^23^, ^24^, ^25^ ^]^ Particularly, *L. monocytogenes* has previously been proven to develop tricks to subvert host mitochondrial network through an inducible and rapid mitochondrial fragmentation in epithelial cells, caused by the precocious release of extracellular LLO.^[^ ^13^, ^14^ ^]^ In this study, we revealed that Mic60, the core component of MICOS, interacts with LLO and is required for *L. monocytogenes*‐induced mitochondrial fragmentation. Additionally, the abundance of host Mic60 was found to contribute an influential role in bacterial cellular infection. Our findings reveal a previously unknown mechanism through which *L. monocytogenes* exploits the MICOS complex subunit Mic60 to modulate mitochondrial dynamics and facilitate its intracellular infection.

**Figure 7:**
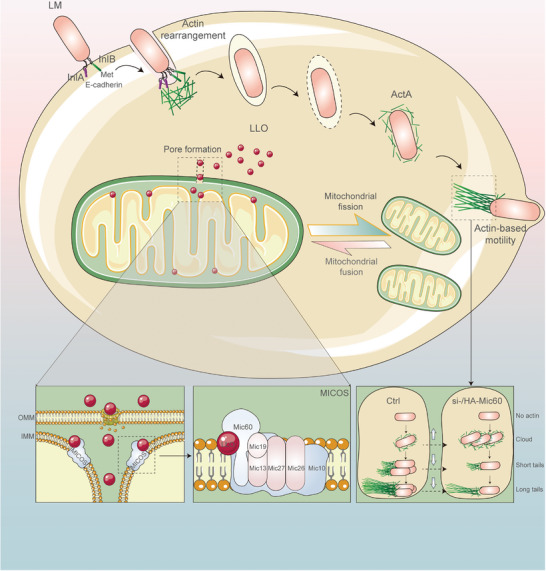
A working model depicting mitochondrial dynamics manipulated by *L. monocytogenes* to favor infection. Upon the entry of *L. monocytogenes* into host cells, the secreted LLO is rapidly transported into the mitochondrial membrane and leads to the OMM permeabilization, thereby facilitating the entry of LLO itself into the IMM. Then the MICOS is targeted by LLO via interactions, which subsequently triggers Mic60‐dependent mitochondrial dynamics and favors bacterial infection.

The mammalian MICOS complex is critical for mitochondrial integrity, consisting of several subunits (Mic10, Mic13, Mic19, Mic25, Mic26, Mic27, and Mic60) that localize predominantly at the cristae junctions of the IMM. These subunits are crucial for maintaining the proper architecture of the IMM.^[^ ^19^, ^26^, ^27^, ^28^ ^]^ Among them, Mic60 is notable for its interactions with both OMM and IMM proteins, playing a dynamic role in mitochondrial structure regulation.^[^ ^19^, ^29^, ^30^, ^31^ ^]^ A recent quantitative mitochondrial proteomics research showed that Mic10 is significantly enriched in mitochondria isolated from cells infected with wild‐type *L. monocytogenes* compared to cells infected with LLO‐deficient bacteria.^[^ ^15^ ^]^ The study revealed that Mic10 is necessary for *L. monocytogenes*‐induced mitochondrial fragmentation.^[^ ^15^ ^]^ Despite this enrichment, we found that Mic10 does not interact with the LLO produced by *L. monocytogenes* (Figure S5, Supporting Information). The molecular basis of this event has pointed to the hypothesis of intramitochondrial Ca^2+^ influx caused by the pore‐forming activity of LLO as a CDC toxin.^[^ ^13^, ^32^ ^]^ Nevertheless, the detailed machinery that mediates the LLO‐dependent mitochondrial fission remains precisely unclear. The study further explores the role of Mic60, an IMM protein that can directly interact with LLO. This interaction suggests a novel mechanism where LLO might penetrate the mitochondrial membrane during bacterial entry, facilitating its access to the IMM. This process differs from the previously understood mechanism where LLO was thought to oligomerize in the plasma membrane, leading to calcium influx.^[^ ^33^, ^34^ ^]^ The interaction between LLO and Mic60 could lead to IMM remodeling and mitochondrial fission, highlighting the strategic targeting of Mic60 by *L. monocytogenes* to manipulate host cell mitochondrial dynamics (**Figure** **7**).

Concerning the differing impacts of Mic10 and Mic60 expression on *L. monocytogenes* proliferation, variations in MICOS component expression influence mitochondrial architecture. Previous research has shown that increased Mic10 levels can substantially extend and deform the cristae structure,^[^ ^35^ ^]^ whereas reduced Mic10 levels lead to a loss of cristae junctions and the stacking of detached cristae within the matrix.^[^ ^17^, ^35^, ^36^ ^]^ The impact of Mic10 on mitochondrial structure distinctly contrasts with that of Mic60. These differential effects of Mic10 and Mic60 expression on mitochondrial architecture might explain their varied impacts on *L. monocytogenes* infection. Cells deficient in Mic60 exhibit significantly altered cristae architecture, resulting in enlarged mitochondria with compromised mitochondrial fusion and fission capabilities.^[^ ^17^, ^20^ ^]^ Overexpression of Mic60 also affects basal mitochondrial dynamics, typically reducing fission and enhancing branching of cristae.^[^ ^18^, ^37^ ^]^ Both downregulation and overexpression of Mic60 influence mitochondrial dynamics in distinct ways but ultimately lead to a reduction in mitochondrial fission and increased ROS production,^[^ ^17^, ^18^, ^20^, ^29^, ^38^ ^]^ supporting our results that both conditions decrease susceptibility to bacterial infection to varying extents.

Bacterial pathogens have evolved diverse mechanisms to manipulate host mitochondrial dynamics to enhance their pathogenic potential. While extracellular bacteria typically introduce effector proteins into host cells while still attached to the cell membrane,^[^ ^23^ ^]^ intracellular bacteria directly interact with mitochondria from within the cytoplasm or vacuoles, such as *Shigella flexneri*,^[^ ^39^ ^]^ *Legionella pneumophila*,^[^ ^40^ ^]^ *Mycobacterium tuberculosis*,^[^ ^41^, ^42^, ^43^ ^]^ and *Salmonella enterica*.^[^ ^44^ ^]^ The *S. flexneri* surface protein IcsA leads to Drp1‐dependent mitochondrial fission in epithelial cells^[^ ^45^ ^]^ and *L. pneumophila* induces mitochondrial fragmentation in macrophages by the secreted MitF, which activates Ran GTPase and triggers Drp1 recruitment.^[^ ^40^ ^]^ Although it is known that these bacteria affect mitochondrial dynamics, the direct mitochondrial targets of their effector proteins remain less defined. Our study, however, sheds light on how *L. monocytogenes*' virulence factors interact with the mitochondrial inner membrane protein Mic60, providing new insights into the mechanisms used by bacterial pathogens to target mitochondrial components.

Another key finding of our study is the discovery of that the precisely‐controlled abundance of endogenous Mic60 correlates positively with intracellular infection of *Listeria* since knockdown or overexpression of Mic60 significantly decreased the susceptibility to bacterial infection. Similarly, Carvalho and co‐workers have previously clarified a role for Mic10, showing that the intracellular *L. monocytogenes* infection efficacy is specifically and positively correlated with increased mitochondrial levels of Mic10.^[^ ^15^ ^]^ However, according to their findings, overexpression of Mic10 showed a slight increase in the levels of intracellular bacteria and none of the other MICOS complex subunits (Mic13, Mic26, and Mic27) are individually required for *L. monocytogenes* cellular infection, while with Mic60 not mentioned.^[^ ^15^ ^]^ Mic60 participates in regulating mitochondrial dynamics via controlling the expression of the other MICOS components as well as the mitochondrial fusion and fission proteins.^[^ ^17^, ^29^, ^46^, ^47^ ^]^ Mutant cells lacking Mic60 display a dramatically altered cristae structure, causing giant mitochondria and impaired mitochondrial fusion and fission.^[^ ^17^, ^35^ ^]^ Similarly, overexpression of Mic60 also influences basal mitochondrial dynamics, favoring decreased fission and increased branching of cristae.^[^ ^18^, ^37^ ^]^ These collectively imply that homeostasis of Mic60 itself determines the mitochondrial dynamics and functions. Besides, Mic60 interacts with all known MICOS components and forms a stable MICOS sub‐complex.^[^ ^46^, ^48^ ^]^ Moreover, the depletion of Mic10 does not affect the protein level of Mic60.^[^ ^17^ ^]^ Hence, it is more likely that Mic60 plays a more critical role than Mic10, or plays independently of Mic10, in the process of facilitating *L. monocytogenes* infection. Also, suppression of Mic60 decreases mitochondrial membrane potential and increases the production of ROS.^[^ ^20^ ^]^ Therefore, modulation of Mic60‐mediated mitochondrial dynamics by *Listeria* allows this bacterium to establish and preserve its intracellular niche to support infection.

Intracellular pathogens must grow inside cells without damaging the host cytoplasmic membrane or triggering the premature death of host cells, with *L. monocytogenes* no exception.^[^ ^49^ ^]^ Pore‐forming toxins LLO is a potent signaling molecule, triggering significant host cell responses via the formation of a large pore complex that allows ions and small molecules to diffuse across the plasma membrane during host infection by the pathogen. To establish a successful infection and achieve maximal virulence, *L. monocytogenes* must maintain an equilibrium between producing LLO that is cytolytic enough to mediate escape from the vacuole and sufficient to trigger a variety of cellular responses, yet is not overly toxic to infected host cells.^[^ ^50^, ^51^ ^]^ Therefore, it has previously been proposed that *L. monocytogenes* requires LLO pore‐forming activity to target mitochondria to induce an intense and rapid but transient fragmentation of the mitochondrial network at early time points of infection in order to establish its replication niche.^[^ ^13^ ^]^ Here, we suggest that *L. monocytogenes* directly targets mitochondria upon infection to enable not only calcium but also LLO influx through pores in the outer mitochondrial membrane formed by the secreted LLO, and then Mic60 is hijacked by this virulence factor to manipulate mitochondrial dynamics.

Our findings highlight a sophisticated mechanism whereby *Listeria* modulates host mitochondrial dynamics, enhancing our understanding of the complex interplay between intracellular bacterial pathogens and host organelles. This research contributes to the broader discourse on the co‐evolution of host‐pathogen interactions and opens avenues for targeted interventions in bacterial infections.

## 4 Experimental Section

### Bacterial Strains and Cell Culture

The wild‐type *L. monocytogenes* strain EGD‐e and its isogenic Δ*hly* mutants (Table S1, Supporting Information) were grown in Brain Heart Infusion medium (BHI, Thermo Fisher Scientific, Waltham, USA) at 37 °C overnight before each experiment unless otherwise specified. Human HeLa and HEK293T cells were cultured in an atmosphere of 5% CO_2_ at 37 °C in Dulbecco's modified Eagle's medium (DMEM, Thermo Fisher Scientific) supplemented with 10% fetal bovine serum (Thermo Fisher Scientific).

### In‐Frame Gene Deletion

The temperature‐sensitive pKSV7 shuttle plasmid was employed for allelic exchange according to the previous method.^[^ ^52^ ^]^ Briefly, the constructed knock‐out plasmid harboring the homologous arms upstream and downstream of the interest gene was electroporated into the competent EGD‐e cells. A single colony of the construct was grown at a non‐permissive temperature (42 °C) on BHI agar containing chloramphenicol to promote chromosomal integration. After then the recombinants were successively passaged without antibiotics at a permissive temperature (30 °C) for enabling plasmid excision and curing. Mutants that lost pKSV7 were identified by sensitivity to chloramphenicol, and finally, allelic exchange was confirmed by PCR and Sanger DNA sequencing when necessary.

### Yeast Two‐Hybrid Screening for PlcB Interactors

Yeast two‐hybrid screening was performed using the Matchmaker Gold Yeast Two‐Hybrid System (Takara, Kusatsu, Japan) to identify interactions between PlcB and host proteins from the Universal Human Mate & Plate Normalized Library (Takara). The bait construct (pGBKT7‐PlcB) was generated and transformed into *S. cerevisiae* strain Y2HGold competent cells and tested for autoactivation and toxicity. The two‐hybrid library between the Y2HGold bait strain and library strain (Y187) was screened using the yeast mating method according to the manufacturer's protocols. Yeast cells were mated, and the colonies were screened on synthetically defined double dropout (DDO/X/A: SD/‐Leu/‐Trp/X‐α‐Gal/AbA) and quadruple dropout (QDO/X/A: SD/‐Ade/‐His/‐Leu/‐Trp/X‐α‐Gal/AbA) medium (Takara). The blue colonies were selected for prey plasmid isolation, and then the prey and bait pGBKT7‐PlcB plasmids were co‐transformed into Y2HGold for verification of genuine interactions in yeast cells. For this experiment, the plasmids pGBKT7‐53 and pGADT7‐T were used as a positive control, and pGBKT7‐Lam and pGADT7‐T were used as a negative control.

### Knockdown and Overexpression of Mic60

To knockdown endogenous host Mic60 gene, 2 × 10^5^ HeLa cells were transfected in a 24‐well plate with appropriate siRNAs (100 nM) using Lipofectamine RNAiMAX (Thermo Fisher Scientific) according to the manufacturer's instructions. The siRNAs Mic60 (5′‐GGUUGUAUCUCAGUAUCAUTT‐3′) and a Universal Negative Control were synthesized, purified, and duplexed by GenoPharma. The efficiency of knockdown of Mic60 was examined 48 h post‐transfection by testing expression levels of Mic60. For transient overexpression of Mic60, HeLa cells were seeded in a 24‐well plate 24 h before transfection with 1 µg of plasmid DNA, using Lipofectamine 2000 (Thermo Fisher Scientific) according to the manufacturer's instructions. The expression plasmids pCMV‐HA‐Mic60 were used to express HA‐tagged Mic60, and cells transfected with pCMV‐HA were used as a negative control. Cells were harvested, and protein expression of Mic60 was assayed 24 h post‐transfection.

### Western Blot Analysis

Cells were harvested, and protein concentrations were quantified using a Bio‐Rad protein assay kit (Beyotime, ShangHai, China). Protein samples were then subjected to SDS‐PAGE and transferred to polyvinylidene difluoride (PVDF) membranes (Millipore, Billerica, USA) using a Semi‐Dry Transfer kit (Bio‐Rad, Hercules, USA). Membranes were blocked using 5% (w/v) non‐fat dry milk and subsequently incubated overnight at 4 °C with the following primary antibodies: mouse monoclonal anti‐c‐Myc (Sigma‐Aldrich, M5546) (1:500), rabbit monoclonal anti‐HA (Cell Signaling Technology, 3724S) (1:1000), mouse monoclonal anti‐Mic60 (Abcam, ab110329) (1:500), and mouse monoclonal anti‐α‐Tubulin (Sigma‐Aldrich, T6199) (1:1000) (Table S1). After incubation with anti‐rabbit or anti‐mouse polyclonal horseradish peroxidase‐labeled secondary antibodies (Abcam, Cambridge, UK), immunoreactive signals were visualized using the enhanced chemiluminescence detection system (UVP Inc., Upland, USA).

### Co‐Immunoprecipitation

HEK293T cells were maintained in DMEM containing 10% FBS. One day before transfection, 1×10^6^ cells were seeded into a well of a 6‐well plate and co‐transfected pCMV‐HA‐Mic60 together with pCMV‐Myc‐LLO (Table S1, Supporting Information)using Lipofectamine 2000 (Thermo Fisher Scientific) according to the manufacturer's protocol. Whole‐cell extracts of transfected cells were prepared 24 hours post‐transfection in cell lysis buffer (Beyotime) supplemented with an EDTA‐free Protease Inhibitor Cocktail (Sangon, Shanghai, China). The supernatant was collected and incubated 6 hours with a mouse monoclonal anti‐c‐Myc antibody (Sigma‐Aldrich) and Dynabeads Protein G (Thermo Fisher Scientific) at 4 °C. Beads were washed five times with the washing buffer (20 mM Tris, 150 mM NaCl, 1% Triton X‐100). Finally, proteins were eluted and dissolved into Laemmli sample buffer containing 5% β‐mercaptoethanol, boiled at 100 °C for 5 min, and subjected to SDS‐PAGE. Immunoblotting was carried out using the rabbit monoclonal anti‐HA antibody (Cell Signaling Technology) and mouse monoclonal anti‐c‐Myc antibody (Sigma‐Aldrich).

### Immunofluorescence Colocalization by Confocal Microscopy

HEK293T cells seeded on coverslips in 24‐well plates were co‐transfected with plasmids pCMV‐HA‐Mic60 and pCMV‐Myc‐LLO. Following 24 h transfection, the cells were washed three times with PBS and fixed with 4% paraformaldehyde (PFA) for 30 min at room temperature, followed by three washes with PBS and permeabilization with 0.1% Triton X‐100 in PBS for 10 min. For colocalization of endogenous Mic60 with LLO, HeLa cells were infected with *L. monocytogenes* at an MOI of 500 for an additional one or five hours after killing extracellular bacteria by gentamicin at one‐hour post‐infection. Cells were fixed as described above and then washed three times with PBS and incubated with the primary antibodies against HA tag (1:500, Cell Signaling Technology), Myc tag (1:500, Sigma‐Aldrich), Mic60 (1:500, Abcam), or LLO (1:1000, this study) diluted in PBS containing 1% BSA for two hours at 37 °C. After additional washing three times with PBS, cells were incubated with the appropriate secondary antibodies (Alexa Fluor 594‐conjugated goat anti‐mouse IgG antibody or Alexa Fluor 488‐conjugated goat anti‐rabbit IgG antibody) (Thermo Fisher Scientific) for one hour at room temperature. Next, the coverslips were washed three times with PBS, counterstained for 5 min with the nuclear stain DAPI (Thermo Fisher Scientific) at room temperature. The coverslips were mounted with the mounting solution (Sigma‐Aldrich), and images were acquired using a confocal laser‐scanning microscope with a 63× objective (Olympus).

### Immunofluorescence of Mitochondrial Dynamics

HeLa cells were seeded at a density of 1 × 10^5^ cells on the coverslips per well of the 24‐well plates the day before infection. Bacterial culture was added to cells at an MOI of 50:1 for one‐hour infection at 37 °C, and cells were washed and incubated for another hour in culture medium supplemented 50 µg mL^−1^ gentamicin, to kill extracellular bacteria. Cells were then fixed with 4% paraformaldehyde (PFA) for 30 min at room temperature, followed by three washes with PBS and permeabilization with 0.1% Triton X‐100 in PBS for 10 min. The anti‐COX IV primary antibody (Cell Signaling Technology, 1:200) in PBS containing 1% BSA was added and incubated for two hours at 37 °C, followed by three washes and incubation with the secondary antibody (Alexa Fluor 594‐conjugated goat anti‐mouse IgG antibody) (Thermo Fisher Scientific) for one hour at room temperature. Next, the coverslips were washed three times with PBS, counterstained for 5 min with the nuclear stain DAPI (Thermo Fisher Scientific) at room temperature. The coverslips were mounted with the mounting solution (Sigma‐Aldrich), and images were acquired using a confocal laser‐scanning microscope with a 63× objective (Olympus). Quantitative analyses of colocalizations were performed with ImageJ software.

### Mitochondrial Morphology Analysis

Quantification of fragmented mitochondria was carried out as previously described,^[^ ^15^, ^53^ ^]^ using the Mitochondrial Network Analysis (MiNA) toolset, a plug‐in within the ImageJ software. Mitochondrial networks from individual cells were selected and digitally isolated before batch analysis. From the output data, we calculated the mitochondrial fragmentation status per cell by using the values listed under “Individuals” (including punctate, rods, and large/round structures) normalized to “Mitochondrial footprint” (mitochondrial area). At least 50 cells were analyzed per experimental condition.

### Intracellular Bacterial Infection and Quantification of Actin Tail Formation

HeLa cells were seeded at a density of 2.5 × 10^5^ cells per well of the 24‐well plates the day before infection. Bacteria were cultured overnight at 37 °C until the exponential phase (OD_600 nm_ of 0.8‐1.0), washed with PBS, and then the bacterial culture was added to cells at a multiplicity of infection (MOI = 50:1). After one‐hour incubation at 37 °C, cells were washed and incubated for another hour at 37 °C in FBS‐containing culture medium supplemented 50 µg mL^−1^ gentamicin to kill extracellular bacteria. For intracellular bacteria counting, cells were then rewashed and incubated in fresh culture medium for an additional four hours (total infection time, six hours). After then, the infected cells were lysed in 500 µL ice‐cold ddH_2_O, and lysates were 10‐fold serially diluted for enumeration of viable bacteria by plating on BHI agar. For observing actin‐tail formation, HeLa cells were infected with GFP‐expressing *L. monocytogenes* at an MOI of 500 for an additional four hours after killing extracellular bacteria by gentamicin. Cells were washed with PBS, fixed with 4% paraformaldehyde and then permeabilized with 0.5% Triton X100. F‐actin was stained with phalloidin‐Alexa Fluor 568 (Thermo Fisher Scientific) and nuclei stained with DAPI (Thermo Fisher Scientific). Actin tails recruited by the bacteria were visualized under a confocal laser‐scanning microscope with a 63× objective (Olympus). Actin tail pattern formation was analyzed by quantifying the percentage of bacteria that displayed long or short actin tails, or were surrounded by F‐actin (clouds), or were not associated with F‐actin. At least 10 microscopy fields were analyzed per experimental condition.

### Transmission Electron Microscopy Analysis

HeLa cells of 2 × 10^6^ were transfected with appropriate siRNAs (100 nM)in a 10 cm cell culture dish using Lipofectamine RNAiMAX (Thermo Fisher Scientific) according to the manufacturer's instructions. Bacterial culture was added to the cells at an MOI of 50:1 for 1 hour infection at 37 °C, and the cells were washed and incubated for a further hour in culture medium supplemented with 50 µg mL^−1^ gentamicin, to kill extracellular bacteria. Cells were then washed three times with cold PBS, scraped and collected by centrifugation at 1000 g for 10 min at 4 °C. Samples were prepared as follows. Sample fixation: cell pellets were suspended and fixed in a solution of 2.5% glutaraldehyde for 24 h at 4 °C, followed by post‐fixation with 1% osmium tetroxide. Sample dehydration: samples were dehydrated in graded series of alcohol (30%, 50%, 70%, 80%), and graded series of acetone (90%, 95%, 100%). Sample infiltration and embedding: samples were placed in graded series of Spurr embedding medium (SPI‐CHEM) in acetone (50%, 75%, 100%), samples were then embedded in Araldite resin and heated at 70 °C overnight. Sample sectioning, staining and observation: samples were then trimmed, sectioned on a LEICA EM UC7 ultramicrotome, and stained with uranyl acetate and lead citrate. Imaging was performed on a JEM‐1010 Jeol (Japan) transmission electron microscope operating at an accelerating voltage of 80 kV.

### Mitochondrial ROS Measurement

Intracellular ROS was measured using the MitoSOX Red (Thermo Fisher Scientific, Waltham, MA, USA) Red. 2 × 10^5^ HeLa cells were transfected with non‐targeting control (si‐Ctrl) or Mic60‐targeting (si‐Mic60) siRNAs, or transiently transfected with the control plasmid (pCMV) or a plasmid constitutively expressing N‐terminal HA‐tagged Mic60 (HA‐Mic60). Then, the Bacterial culture of wild‐type *L. monocytogenes* and △*hly* was then added to the cells at an MOI of 50:1 for 1 hour infection at 37 °C, and the cells were washed and incubated for a further hour in culture medium supplemented with 50 µg mL^−1^ gentamicin, to kill extracellular bacteria. After treatment, the cells were collected from the cell culture plates and centrifuged at 700 rpm for 3 minutes, the collected cells were washed twice with PBS and incubated with 5 µM MitoSOX Red reagent in serum‐free DMEM at 37 °C for 30 minutes. The cell pellet was collected after centrifugation, washed twice with PBS, and finally resuspended in 500 µL PBS for flow cytometry analysis.

### Mitochondrial Membrane Potential Measurement

Intracellular mitochondrial membrane potential was measured using the Mitochondrial membrane potential assay Kit with JC‐1 (Solarbio). HeLa cells of 2 × 10^5^ were transfected with non‐targeting control (si‐Ctrl) or Mic60‐targeting (si‐Mic60) siRNAs, or transiently transfected with the control plasmid (pCMV) or a plasmid constitutively expressing N‐terminal HA‐tagged Mic60 (HA‐Mic60). Bacterial culture was then added to the cells at an MOI of 50:1 for 1 hour infection at 37 °C, and the cells were washed and incubated for a further hour in culture medium supplemented with 50 µg mL^−1^ gentamicin, to kill extracellular bacteria, followed by incubation with JC‐1 working solution for 20 minutes at 37 °C. Mean fluorescence intensity was determined using a Synergy H1 microplate reader (BioTek) with Em/Ex = 590/525 nm to detect red fluorescence and Em/Ex = 530/490 nm to detect green fluorescence.

### Mitochondria Isolation and Co‐Immunoprecipitation of Mitochondrial Protein

THP‐1 cells were maintained in 1640 with 10% FBS. One day before transfection, 1×10^7^ cells were seeded into three T75 cell culture flasks and phorbol 12‐myristate 13‐acetate (sangon) was added at a final concentration of 100 ng ml^−1^. The T75 cell culture flasks were placed in a 5% CO_2_ cell incubator at 37 °C for 24 hours to allow the cells to adhere to the flask wall. THP‐1 cells were then infected with *L. monocytogenes* EGD‐e for 3 hours (MOI = 10). Mitochondria were then extracted from the infected cells using the Cell Mitochondria Isolation Kit (Beyotime) according to the manufacturer's protocol. Briefly, the collected cells were first resuspended with mitochondrial isolation reagent supplemented with the protease inhibitor phenylmethylsulfonyl fluoride (PMSF), then the cell suspension was transferred to a glass homogenizer for homogenization and centrifuged to collect mitochondria, and finally mitochondrial lysis buffer supplemented with PMSF was added. The supernatant was collected and were added with 5 µL Dynabeads Protein G (Thermo Fisher Scientific) and bound for 3 hours at 4 °C to prevent nonspecific binding. The supernatant was collected by centrifugation, and the rabbit polyclonal anti‐Mic60 antibody (Abcam) was added to bind overnight at 4 °C, then 10 µL beads were added and bound for 4 hours at 4 °C. The beads were washed seven times with wash buffer. Finally, the proteins were eluted and solubilized and subjected to SDS‐PAGE. Immunoblotting was carried out using the rabbit polyclonal anti‐Mic60 antibody, the rabbit polyclonal anti‐LLO antibody (Lab stock), and the mouse monoclonal anti‐COX IV antibody (Cell Signaling Technology). COX IV was used as an internal control for mitochondrial protein.

### Quantification and Statistical Analysis

All results were presented as the average ± standard error of the mean. Data were analyzed using the two‐tailed homoscedastic Student's t‐test. A *p*‐value ≤ 0.05 was considered as statistically significant.

### Ethics Statement

All animal experimentation was approved by the Institutional Animal Care and Use Committee of Science Technology Department of Zhejiang Province (Permit Number: SYXK‐2023‐0015) in accordance with the Regulations for the Administration of Affairs Concerning Experimental Animals.

## Conflict of Interest

The authors declare no conflict of interest.

## Author Contributions

C.C., H.S., and W.F. conceived and designed the experiments. C.C., M.C., J.S., J.X., S.D., J.X., Y.H., X.Z., J.W., L.L., R.Z. and Q.W. performed the experiments. C.C., M.C., and J.S. analyzed the data. C.C. wrote the paper.

## Supporting information



c

## Data Availability

The data that support the findings of this study are available in the supplementary material of this article.
