# Supplementary material for: The MICOS Complex Subunit Mic60 is Hijacked by Intracellular Bacteria to Manipulate Mitochondrial Dynamics and Promote Bacterial Pathogenicity
Source: Adv Sci (Weinh). 2024 Oct 21;11(46):2406760. doi: 10.1002/advs.202406760 (PMC11633497; doi:10.1002/advs.202406760)
Supplement: Supplementary file 1 — c [file ADVS-11-2406760-s001.docx]

**Supplemental Information for:**

**The MICOS complex subunit Mic60 is hijacked by intracellular bacteria to manipulate mitochondrial dynamics and** **promote bacterial pathogenicity**

Changyong Cheng, Mianmian Chen, Jing Sun, Jiali Xu, Simin Deng, Jing Xia, Yue Han, Xian Zhang, Jing Wang, Lei Lei, Ruidong Zhai, Qin Wu, Weihuan Fang, Houhui Song*

Key Laboratory of Applied Technology on Green-Eco-Healthy Animal Husbandry of Zhejiang Province, Zhejiang Provincial Engineering Research Center for Animal Health Diagnostics & Advanced Technology, Zhejiang International Science and Technology Cooperation Base for Veterinary Medicine and Health Management, China-Australia Joint Laboratory for Animal Health Big Data Analytics, College of Animal Science and Technology & College of Veterinary Medicine of Zhejiang A&F University, 666 Wusu Street, Lin’an District, Hangzhou, Zhejiang Province 311300, China.

*Correspondence: [songhh@zafu.edu.cn](mailto:songhh@zafu.edu.cn)

**Table S1** Key resource in this study

**Figure S1** Mic60 was screened for interaction with PlcB by using the yeast two-hybrid system

**Figure S2** Mic60 was identified as a target interacting with LLO in host mitochondria infected by *L. monocytogenes*

**Figure S3** Mic60 overexpression blocks *L. monocytogenes* infection-induced mitochondrial fragmentation

**Figure S4** Mitochondrial morphology, mitochondrial membrane potential loss and mitochondrial ROS production in Mic60 knockdown, Mic60 overexpression and *L. monocytogenes* -infected cells.

**Figure S5** Mic10 does not interact with LLO

**Table S1 Key resource in this study**

| **REAGENT or RESOURCE** | **SOURCE** | **IDENTIFIER** |
| --- | --- | --- |
| Antibodies | | |
| Mouse monoclonal anti-c-Myc antibody produced | Sigma-Aldrich | Cat#M5546; RRID:AB_260581 |
| Mouse monoclonal anti-α-Tubulin antibody | Sigma-Aldrich | Cat#T9026; RRID:AB_477593 |
| Mouse monoclonal anti-Mitofilin-Mitochondrial Marker | Abcam | Cat#ab110329; RRID:AB_10859613 |
| [Mouse monoclonal control IgG](https://abclonal.com.cn/catalog/AC011) | ABclonal | [RRID:AB_2770414](http://antibodyregistry.org/AB_2770414) |
| Rabbit monoclonal anti-HA-Tag | Cell Signaling Technology | Cat#3724S; RRID:AB_1549585 |
| Mouse monoclonal anti-COX IV | Cell Signaling Technology | Cat#11967S; RRID:AB_2797784 |
| Goat anti-Mouse IgG (H+L) Highly Cross-Adsorbed Secondary Antibody, Alexa Fluor 594 | Thermo Fisher Scientific | Cat#A11032; RRID:AB_2534091 |
| Donkey anti-Rabbit IgG (H+L) Highly Cross-Adsorbed Secondary Antibody, Alexa Fluor 488 | Thermo Fisher Scientific | Cat#A21206; RRID:AB_2535792 |
| Bacterial Strains | | |
| *Listeria monocytogenes*: EGD-e | Lab stock | Lm-3, from Nancy Freitag |
| *Listeria monocytogenes*: EGD-e Δ*hly* | Lab stock | Lm-156 |
| *Listeria monocytogenes*: EGD-e-GFP | Lab stock | Lm-48 |
| *Listeria monocytogenes*: EGD-e *hly_F251Y_* | Lab stock | Lm-1860 |
| *Listeria monocytogenes*: EGD-e *hly*_Δ251-255_ | Lab stock | Lm-1862 |
| Chemicals | | |
| Cell lysis buffer for Western and IP | Beyotime | Cat#P0013 |
| Fetal Bovine Serum, qualified, heat inactivated, Australia | Thermo Fisher Scientific | Cat#10100147 |
| DMEM, high glucose, pyruvate | Thermo Fisher Scientific | Cat#11995073 |
| Trypsin-EDTA (0.25%), phenol red | Thermo Fisher Scientific | Cat#25200072 |
| DAPI | Thermo Fisher Scientific | Cat#1217226 |
| [Alexa Fluor™ 594 Phalloidin](https://www.thermofisher.com/order/catalog/product/A12381?SID=srch-srp-A12381) | Thermo Fisher Scientific | Cat#A12381 |
| Critical Commercial Assays | | |
| Dynabeads™ Protein G for Immunoprecipitation | Thermo Fisher Scientific | Cat#10004D |
| Lipofectamine™ RNAiMAX Transfection Reagent | Thermo Fisher Scientific | Cat#13778075 |
| Lipofectamine™ 2000 Transfection Reagent | Thermo Fisher Scientific | Cat#11668019 |
| pCMV-Myc & pCMV-HA Vector Set | Clontech | Cat#631604 |
| KOD OneTM PCR Master Mix | TOYOBO | Cat#KMM-201 |
| Experimental Models: Cell Lines | | |
| Human: HeLa cell line | Lab stock | ATCC® CCL-2™ |
| Human: HEK293T cell line | Lab stock | ATCC® CRL-11268™ |
| Oligonucleotides | | |
| Mic60 siRNA targeting sequence: GGUUGUAUCUCAGUAUCAUTT | Gene pharma | <http://www.genepharma.com/> |
| Recombinant DNA | | |
| pCMV-HA-Mic60_34-758_ | This study | pSL2401 |
| pCMV-HA-Mic60_1-758_ | This study | pSL2413 |
| pCMV-Myc-LLO_26-480_ | This study | pSL1941 |
| pCMV-Myc-LLO_26-420_ | This study | pSL2201 |
| pCMV-Myc-LLO_26-360_ | This study | pSL2203 |
| pCMV-Myc-LLO_26-330_ | This study | pSL2427 |
| pCMV-Myc-LLO_26-315_ | This study | pSL2429 |
| pCMV-Myc-LLO_26-300_ | This study | pSL2204 |
| pCMV-Myc-LLO_26-280_ | This study | pSL2215 |
| pCMV-Myc-LLO_26-255_ | This study | pSL2228 |
| pCMV-Myc-LLO_26-250_ | This study | pSL2224 |
| pCMV-Myc-LIO_26-480_ | This study | pSL2235 |
| pCMV-Myc-LSO_26-481_ | This study | pSL2236 |
| pCMV-Myc-PFO_26-455_ | This study | pSL2231 |
| pCMV-Myc-LLO_F251Y_ | This study | pSL2432 |
| pCMV-Myc-LLO_Y255F_ | This study | pSL2434 |
| pCMV-Myc-PFO_Y226F_ | This study | pSL2435 |
| pCMV-Myc-PFO_F230Y_ | This study | pSL2436 |
| Software and Algorithms | | |
| ImageJ | NIH | https://imagej.nih.gov/ij/ |
| Prism8 | GraphPad | <https://www.graphpad.com/> |
| MINA | [Andrew J.Valente](https://www.sciencedirect.com/science/article/pii/S0065128116303658?via%3Dihub#!) | <https://doi.org/10.1016/j.acthis.2017.03.001> |
| Others | | |
| Olympus FV3000 epifluorescence microscope | Olympus | <https://www.olympuslifescience.com.cn/laser-scanning/fv3000/> |

**Figure S1**


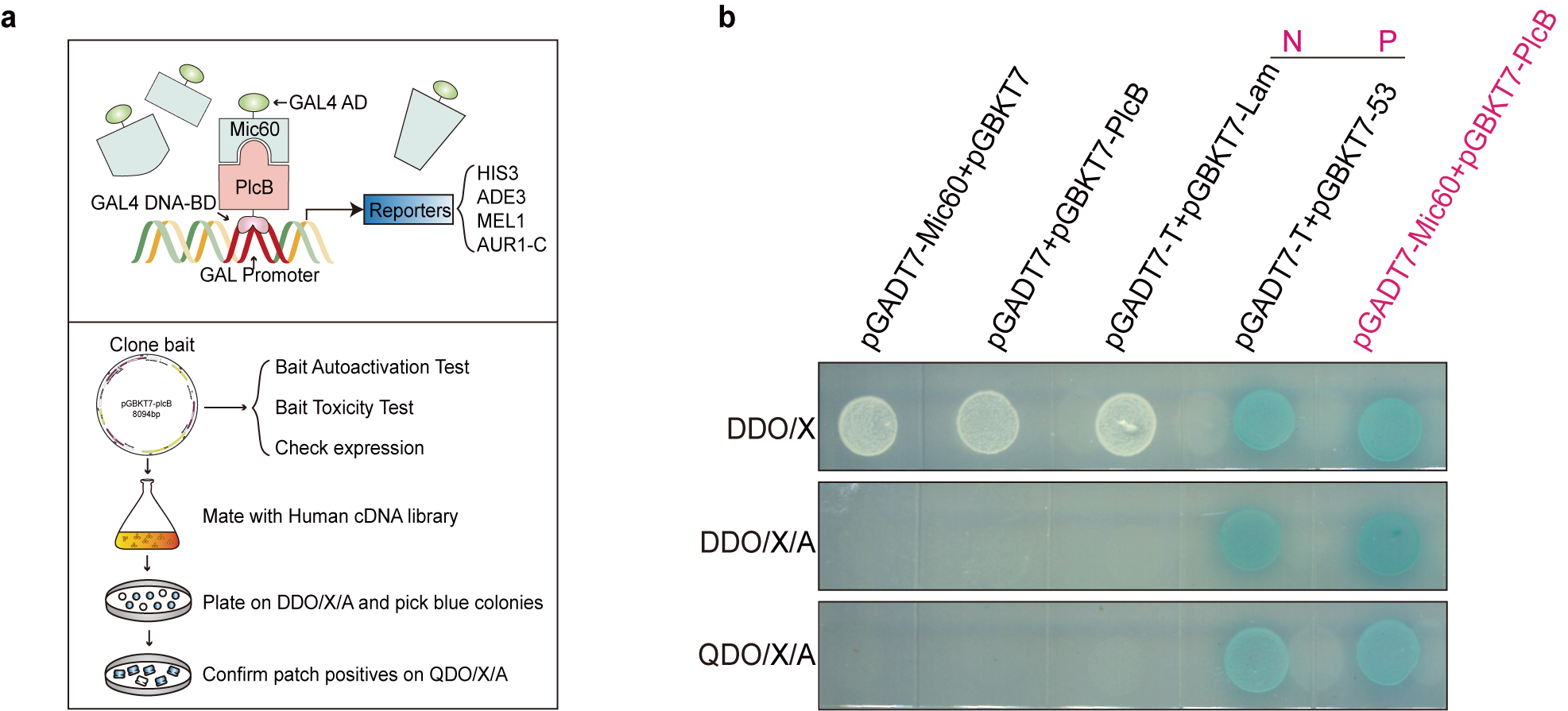


**Fig. S1** **Mic60 was screened for interaction with PlcB by using the yeast two-hybrid system**

**a** Strategy for screening host proteins interacting with PlcB by using the yeast two-hybrid system (Y2H). Interaction of PlcB and human Mic60 proteins reconstitutes the function of the Gal4 protein and results in the expression of the reporter genes, *AUR1-C, ADE2, HIS3, and MEL1*.

**b** α-galactosidase activity of yeast cells expressing various combinations of BD and AD fusions to verify protein interactions. The co-transformed yeast cells were grown on the selective double dropout (DDO/X/A: SD/-Leu/-Trp/X-α-Gal/AbA) and quadruple dropout (QDO/X/A: SD/-Ade/-His/-Leu/-Trp/X-α-Gal/AbA) medium. The blue colonies were expected as genuine interactions in yeast cells. For this experiment, the combination of plasmids pGBKT7-53 and pGADT7-T was used as a positive control (P), and combination of pGBKT7-Lam and pGADT7-T was used as a negative control (N).

**Figure S2**


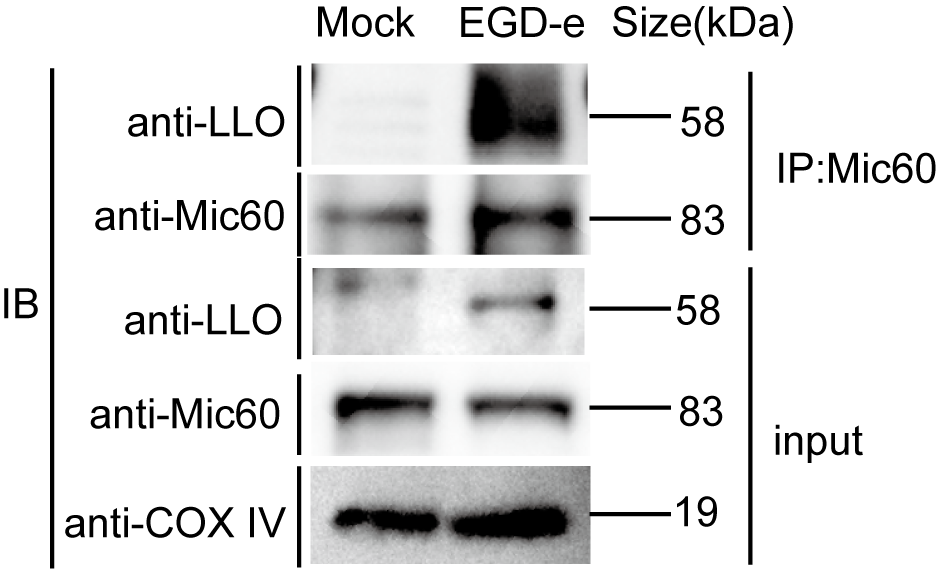


**Fig. S2** **Mic60 was identified as a target interacting with LLO in host mitochondria infected by *L. monocytogenes***

THP-1 cells were infected with *L. monocytogenes* EGD-e for 3 hours (MOI=10). Mitochondria were then extracted from the infected cells using the Cell Mitochondria Isolation Kit according to the manufacturer's protocol, and mitochondrial protein sample was obtained when mitochondrial lysis buffer was added. Co-immunoprecipitation of LLO with Mic60 from mitochondrial protein sample using an anti-Mic60 antibody. Subsequent immunoblotting was performed using antibodies against LLO and Mic60. COX IV was used as an internal control for mitochondrial protein.

**Figure S3**

**
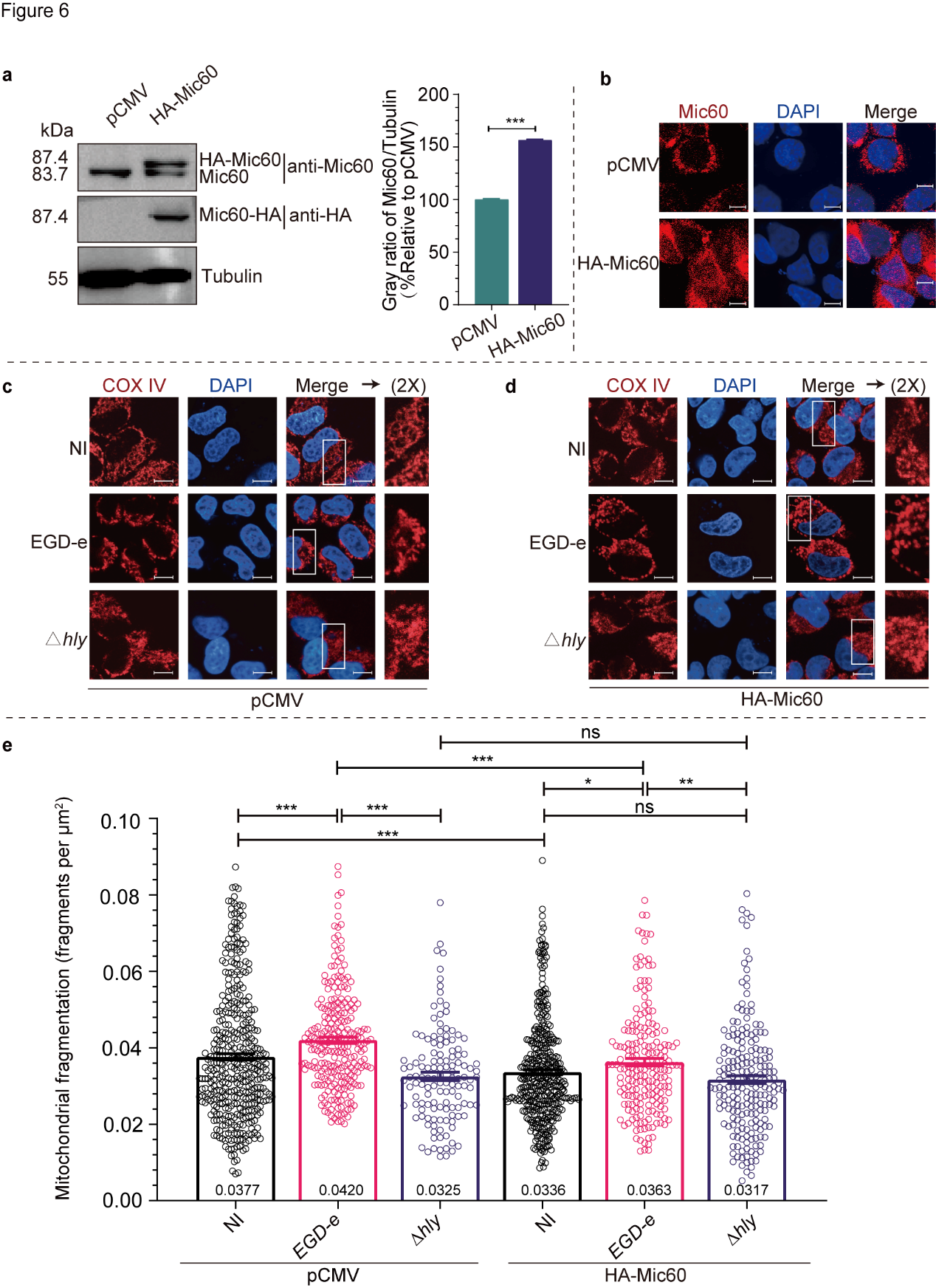
**

**Fig. S3 Mic60 overexpression blocks *L. monocytogenes* infection-induced mitochondrial fragmentation**

**a** Immunoblotting analysis of Mic60 levels in HeLa cells transfected with a control plasmid (pCMV) or a plasmid constitutively expressing HA-tagged Mic60 (Mic60-HA). The endogenous Mic60 was detected with an anti-Mic60 antibody, and exogenous Mic60-HA detected simultaneously with anti-Mic60 and anti-HA antibodies. Tubulin protein was used as the internal control. The histogram on the right indicates the grayscale ratio of Mic60 to tubulin, with the control group set to 100%. Data are expressed as means ± SE of three independent experiments. **, *p* < 0.01.

**b** Immunofluorescence analysis of HeLa cells transiently transfected with pCMV or HA-Mic60 plasmids. Mic60 proteins are shown in red (anti-Mic60) and nuclei in blue (DAPI). Scale bars, 10 μm.

**c-d** Immunofluorescence analysis of HeLa cells transfected with pCMV (c) or HA-Mic60 (d) plasmids, which were infected for 2 h with wild-type, △*hly* *L. monocytogenes* at an MOI of 50. Mitochondria are shown in red (COX IV) and nuclei in blue (DAPI). The white box indicates a region of the mitochondrial network magniﬁed (2x) in the inset shown at the right. NI, no infection; Scale bars, 10 μm.

**e** Quantiﬁcation for the degree of mitochondrial fragmentation in HeLa cells infected with *L. monocytogenes*. Scatter plots show fragmentation degree values for each cell (at least 50 dots), and data are expressed as means ± SE of three independent experiments. NI, no infection; ns, not significant; ***, *p* < 0.001.

**Figure S4**

**
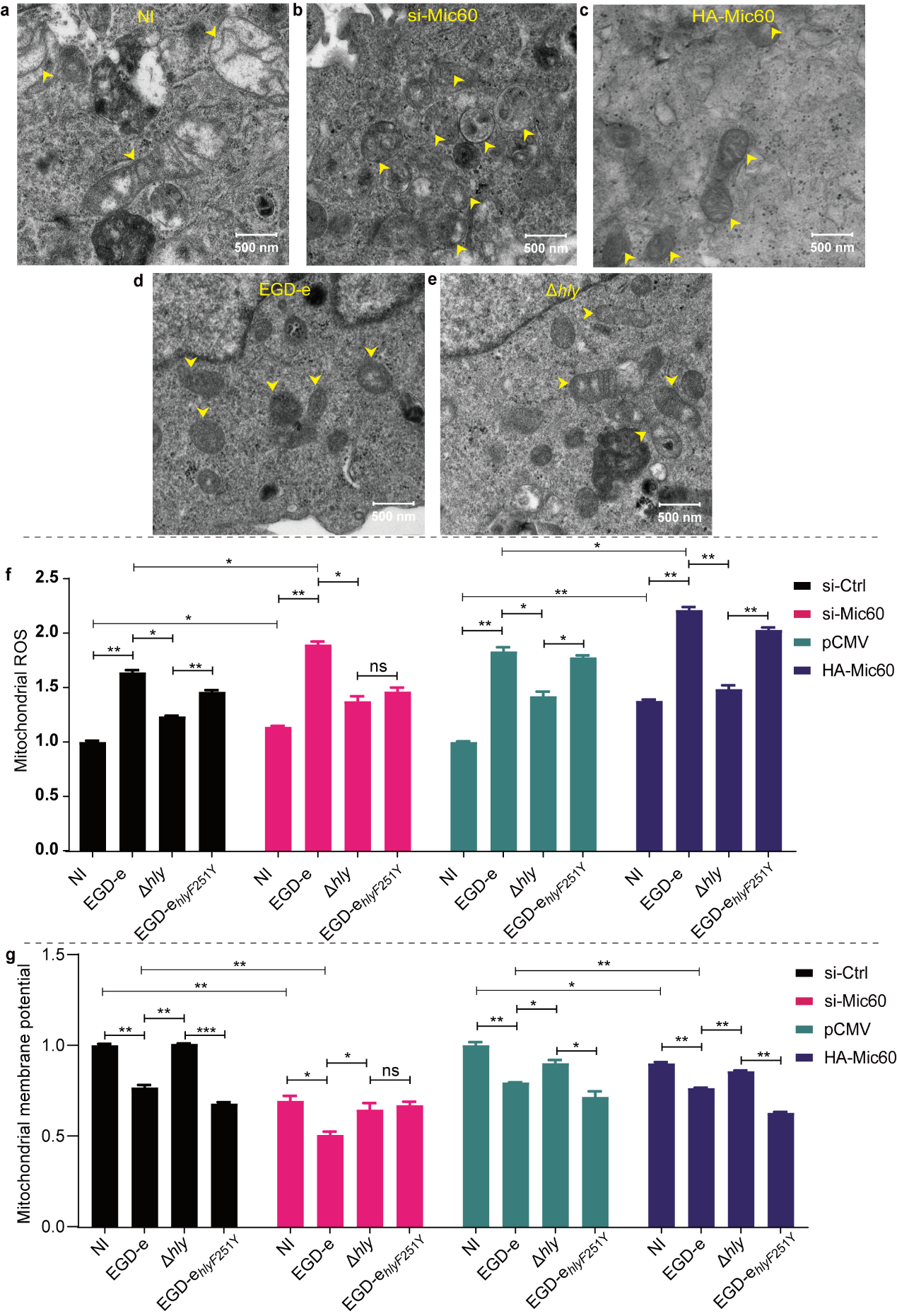
**

**Fig. S4 Mitochondrial morphology, mitochondrial membrane potential loss and mitochondrial ROS production in Mic60 knockdown, Mic60 overexpression and *L. monocytogenes*-infected cells.**

**a-e** Electron micrographs of mitochondria in Hela cells without infection (a), Mic60 knockdown cells (b), Mic60 overexpression cells (c), *L. monocytogenes* WT-infected Hela cells (d), and △*hly*-infected Hela cells (e).

**f** HeLa cells transfected with non-targeting control (si-Ctrl) or Mic60-targeting (si-Mic60) siRNAs, or transiently transfected with the control plasmid (pCMV) or a plasmid constitutively expressing N-terminal HA-tagged Mic60 (HA-Mic60) were infected with wild-type *L. monocytogenes*, △*hly* and *hly*_F251Y_ for 2 h, followed by incubation with 5 μM MitoSOX Red for 30 min at 37°C. The cell pellet was collected after centrifugation, washed twice with PBS, and finally resuspended in 500 μL PBS for flow cytometry analysis.

**g** HeLa cells transfected with non-targeting control (si-Ctrl) or Mic60-targeting (si-Mic60) siRNAs, or transiently transfected with the control plasmid (pCMV) or a plasmid constitutively expressing N-terminal HA-tagged Mic60 (HA-Mic60) were infected with wild-type *L. monocytogenes*, △*hly* and *hly*_F251Y_ for 2 h, followed by incubation with JC-1 working solution for 20 min at 37°C. Mean fluorescence intensity was determined using a Synergy H1 microplate reader (BioTek) with Em/Ex=590/525 nm for detection of red fluorescence and Em/Ex=530/490 nm for detection of green fluorescence.

**Figure S5**


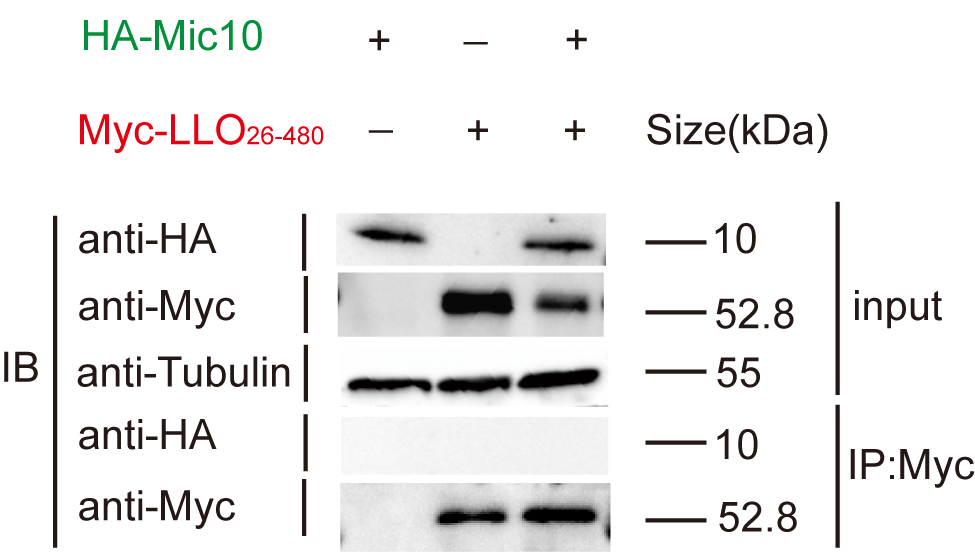


**Fig. S5 Mic10 does not interact with LLO**

Co-immunoprecipitation of HA-Mic10 with Myc-LLO from co-transfected HEK293T cells using an anti-Myc antibody. Transfected with one eukaryotic expression plasmid was used as negative control for immunoprecipitation. Subsequent immunoblotting was performed using antibodies against HA and Myc tags. Tubulin protein was used as the internal control.
